# Polyhedral Oligomeric Silsesquioxane (POSS)-Containing Polymer Nanocomposites

**DOI:** 10.3390/nano2040445

**Published:** 2012-12-06

**Authors:** Ebunoluwa Ayandele, Biswajit Sarkar, Paschalis Alexandridis

**Affiliations:** Department of Chemical and Biological Engineering, University at Buffalo, The State University of New York (SUNY), Buffalo, NY 14260-4200, USA; E-Mails: eaa23@buffalo.edu (E.A.); palexand@buffalo.edu (P.A.)

**Keywords:** inorganic-organic nanocomposites, polyhedral oligomeric silsesquioxane (POSS), hybrid polymer, mechanical properties, thermal stability

## Abstract

Hybrid materials with superior structural and functional properties can be obtained by incorporating nanofillers into polymer matrices. Polyhedral oligomeric silsesquioxane (POSS) nanoparticles have attracted much attention recently due to their nanometer size, the ease of which these particles can be incorporated into polymeric materials and the unique capability to reinforce polymers. We review here the state of POSS-containing polymer nanocomposites. We discuss the influence of the incorporation of POSS into polymer matrices via chemical cross-linking or physical blending on the structure of nanocomposites, as affected by surface functional groups, and the POSS concentration.

## 1. Introduction

Polymers are widely used in industry due to their light weight and ductility. However, they have a lower modulus and strength when compared to metals and ceramics [1], which may make polymers less attractive as engineering materials. An effective way to improve these mechanical properties is by reinforcing the polymer matrix with fillers, such as fibers, platelets or nanoparticles. However, enhancement of a certain physical property of a polymer by incorporating macro-sized fillers often involves a trade-off [2]. For example, the stiffness of a polymer can be increased by incorporating suitable macro-sized fillers, but the resulting composite would lose its toughness. Polymer nanocomposites, a new class of hybrid materials composed of polymers into which nano-sized inorganic particles (1–100 nm [3,4,5,6]) are dispersed, can produce desirable structural and functional properties without compromising other valuable properties. Nanoparticles impart functional properties, and polymers provide structure and processability. Nanometer-sized filler materials have a large surface-area-to-volume ratio and are of particular interest, because they can be easily dispersed in a polymer, hence facilitating the enhancement of a desired property, such as modulus, strength, heat resistance, porosity (barrier property) and flammability [3,5,7,8].

**Figure 1:**
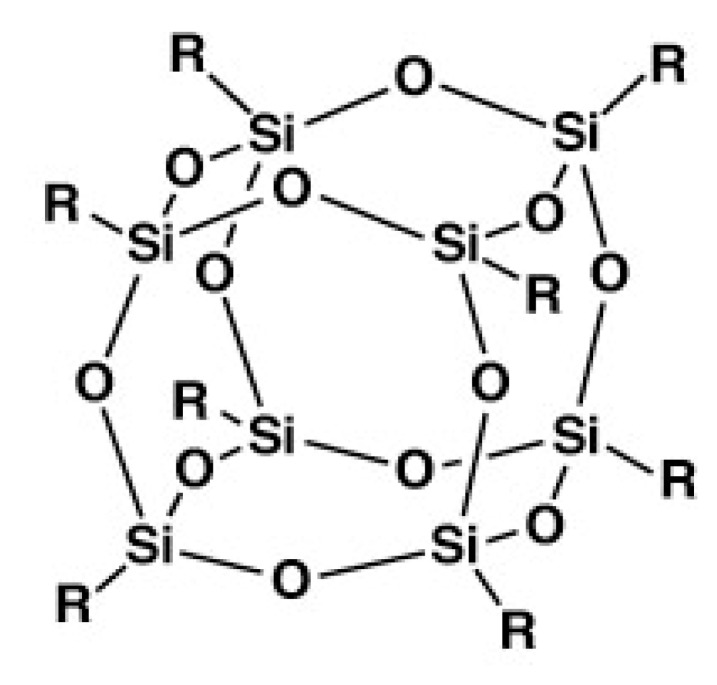
Molecular structure of polyhedral ologomeric silsesquioxane (POSS).

Polyhedral oligomeric silsesquioxanes (POSS) are nanostructures with the empirical formula RSiO_1.5_, where R may be a hydrogen atom or an organic functional group, e.g., alkyl, alkylene, acrylate, hydroxyl or epoxide unit [9,10,11,12]. POSS may be referred to as a silica nanoparticle consisting of a silica cage core, as well as other organic functional groups attached to the corners of the cage (Figure 1) [13]. POSS consists of both organic and inorganic matter with an inner core of inorganic silicon and oxygen and an outer layer of organic constituents, which could be either polar or nonpolar. POSS can be divided into molecular silica, monofunctional POSS and multifunctional POSS [9]. When all the organic groups are non-reactive, they are referred to as molecular silica. If one of the organic groups is reactive, these POSS are called monofunctional POSS or MonoPOSS. If more than one of the organic groups is reactive, they are known as multifunctional POSS. POSS molecules whose organic groups are all reactive are frequently encountered in the multifunctional POSS category. POSS with different R-groups and their properties are reviewed in [10]. Silsesquioxanes structures can be random, ladder, cage or partial cage [9,10,14].

POSS nanostructures have diameters in the range 1–3 nm and, hence, may be considered as the smallest existing silica particles [9]. POSS particles have been classified as having a zero-dimensionality; however, the ability to create higher dimensionality (1, 2 or 3-D scaffolds) through aggregation or crystallization of the POSS particles within the polymer matrix has been reported [15]. This ability of POSS to serve as building blocks plays a key role in motivating the study of POSS in polymer matrices.

POSS nanoparticles can be used to decrease viscosity in highly filled resins by forming strong bonds with the surface of the fillers and breaking up particle-particle interactions, resulting in enhanced mechanical properties and surface finish [16]. Unlike most fillers, POSS molecules contain organic constituents on their external surface, which can make them compatible with many polymers [9]. Researchers have found that dispersing POSS nanoparticles into a polymer increases the strength, modulus, rigidity and reduces the flammability, heat discharge and viscosity of the polymer [1,3,17,18], while retaining its lightweight and ductile features. These enhanced properties allow for a wider range of applications of these nanocomposites, e.g., drug delivery, polymer electrolytes, thermoplastic and thermosetting polymers [19,20,21,22]. Other benefits to POSS include being non-volatile, odorless and overall environmentally friendly. In addition, the ease with which they can be synthesized makes them commercially available [9]. Considering these potential commercial uses and their increased performance over their non-hybrid counter parts, POSS-containing polymer nanocomposites have been widely investigated [9,10,23,24,25,26,27,28,29,30,31].

Properties of POSS-containing polymer composites depend on the successful incorporation of POSS particles in polymeric matrices. Two approaches have been adopted to incorporate POSS particles into polymer matrices: (i) chemical cross-linking [9,32] and (ii) physical blending. In the first approach, POSS nanoparticles are bonded covalently with polymer. In the second approach, POSS nanoparticles are physically blended with polymer by melt mixing or solvent casting methods. The success of physical blending depends on the compatibility of POSS particles with the polymers [33,34]. Interactions between nanoparticle and polymer are mediated by ligands, which are attached to the nanoparticles, and hence, ligands play a remarkable role in influencing the particle behavior and spatial distribution [4]. The surface functionalization of nanoparticles can be achieved by attaching small functional molecules and polymers either covalently [4] or by physical adsorption [35,36]. There are several key challenges encountered in preparing POSS-containing polymer-nanocomposites, some of which include long range equilibration time, aggregation of nanoparticles, and expensive large-scale production [4,37]. The control of nanostructure and location of nanoparticles in polymer-nanocomposites remain open challenges. As a result, processing methods have been developed to incorporate nanoparticles into polymer matrices either by *in situ* polymerization or by physical blending (e.g., melt mixing).

Recently, issues related to structure and functional properties of nanoparticle-containing (particularly POSS-containing) polymer nanocomposites have attracted our attention, along with many other research groups. We have studied block copolymer-nanoparticle composite formation by synthesizing nanoparticles *in situ *[38,39,40,41]. We also have addressed thermodynamic issues related to incorporation of nanoparticles in solvated block copolymer nanostructures, where nanoparticles were synthesized *ex situ* and were templated by solvated block copolymers [42,43,44]. Surface interactions of amphiphilic block copolymer with nanoparticles have also been studied [45,46]. Currently, we are working on block copolymer nanocomposites incorporating POSS particles of varying surface functionalities.

The focus of this review is to present an account of fundamental understanding of structure and functional properties of POSS-containing polymer nanocomposites. We have considered the incorporation of POSS nanoparticles into polymeric matrices via chemical cross-linking or physical blending. In Part 2, we discuss the influence of chemically cross-linked POSS on the structure of POSS-containing polymeric composites, considering examples of various polymeric systems. Similarly, in Part 3, we discuss the influence of the incorporation of POSS in polymeric matrices (via physical blending) on the overall structure, particularly, how POSS-polymer surface interactions (as affected by the surface functional group of POSS and loading) influence the nanocomposite structure. In Part 4, we discuss the influence of POSS incorporation in polymer on functional properties such as mechanical, thermal stability, and glass transition. Specific examples are given to emphasize the role of POSS and their state of dispersion-influenced functional properties of POSS-containing polymeric composites.

## 2. Chemically Cross-Linked POSS-Containing Polymer Composites

In past reports on the preparation of POSS-polymer hybrids, POSS molecules were mostly used to enhance polymer properties by incorporating them into the polymer matrix via chemical copolymerization, cross-linking or mere physical blending [9,47]. One major difference between physical blending and polymerization is that the macroscopic phase separation between the POSS particles and the polymer matrix that may occur in the former is absent in the latter due to covalent bonding in the POSS copolymers [14]. POSS-based block copolymers synthesized via physical blending are discussed in section 3. This section discusses POSS-containing polymers nanocomposites, where POSS particles are incorporated via chemical cross-linking with the polymer.

### 2.1. POSS-Styrene Based Nanocomposites

Incorporation of POSS into styrene-based polymers was found to influence the microstructure of the composites. POSS has been incorporated into cylindrical structures formed by poly(styrene)-poly(butadiene)-poly(styrene) (SBS) triblock copolymer through the hydrosilylation technique [48]. POSS particles containing a silane functional group were grafted covalently onto poly(butadiene) (PB) blocks. This investigation revealed that the SBS cylindrical structure lost its long range order due to the incorporation of POSS. To study further the role of particle-polymer surface interactions, POSS particles with different surface functional groups (POSS molecules contain one silane, which can be used to graft onto poly(butadiene) blocks and seven organic functional groups of identical nature) were synthesized [49,50]. Grafting of phenyl functionalized POSS onto PB blocks led to a decrease in the lattice parameter of the SBS microstructure, as well as the order to disorder transition (ODT) temperature [50]. Phenyl groups had cooperative surface interactions with poly(styrene) (PS) blocks, which decreased the degree of block segregation. Replacement of phenyl functionalized POSS with isobutyl (iBu) functionalized POSS was found not to influence the lattice parameter, long range order and ODT [49]. iBu groups did not interact with PS blocks, and therefore, grafting of iBu-functionalized POSS on the PB blocks did not influence the degree of block segregation. Thus, the compatibility of the POSS with the host polymer plays a very important role.

### 2.2. POSS-Methacrylate-Based Nanocomposites

**Figure 2:**
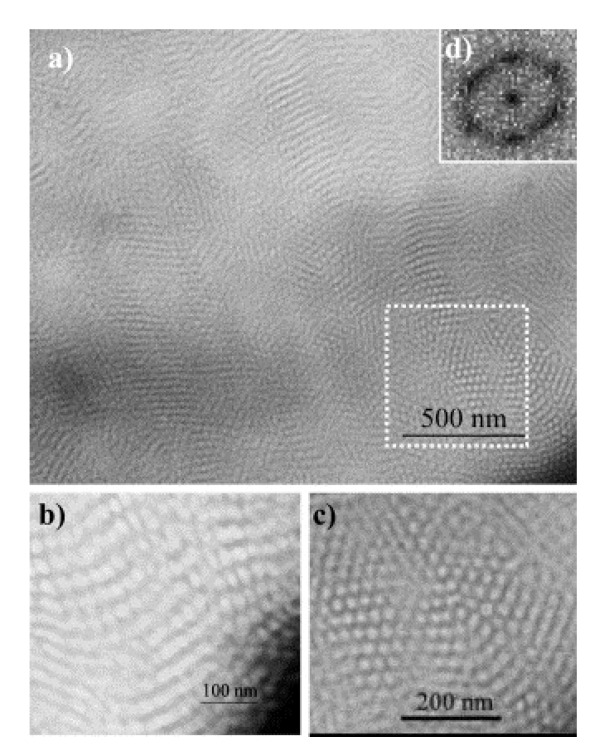
Transmission electron microscopy (TEM) of RuO_4_ treated cryo-microtomed (*T* = −80 °C) thin sections of p(MA-POSS)_10_-pBA_201_-p(MA-POSS)_10_ triblock copolymer nanocomposites: (**a**) overall morphology; (**b**,**c**) cylindrical morphology; and (**d**) Fourier transform of the area are enclosed in dotted lines. Reproduced with permission from Pyun *et al. *[51], Copyright 2003, Elsevier.

Methacrylate (MA)-based polymers (such as poly(methyl methacrylate), PMMA) find many applications because of ease of processing and high modulus. Lower glass transition temperature (Tg) and poor thermal stability limit many applications of PMMA-based polymers. In order to improve those properties, POSS was introduced to polymer backbone covalently via free radical polymerization (FRP) or via atom transfer radical polymerization (ATRP). Pyun *et al. *[51] synthesized POSS-containing ABA triblock copolymers possessing a soft poly(*n*-butyl acrylate) (pBA) middle segment and glassy poly(MA-POSS) (p(MA-POSS)) outer segments. Phase separated microstructures were formed in thin films of the copolymer by tuning the relative composition and the degree of polymerization of both segments. Transmission electron microscopy (TEM) revealed that for a small concentration of p(MA-POSS), there was no evidence of microphase separation, while a high p(MA-POSS) concentration resulted in strong microphase separation (Figure 2). Dynamic mechanical analysis (DMA) revealed that the microphase-separated material exhibited a higher tensile modulus than expected. More about the thermo-mechanical properties for this material is discussed in section 4. Successful incorporation of octavinyl-POSS into poly(methyl-MA) (PMMA) polymer was found to be effective in improving the Tg of the polymer nanocomposite [52].

Hirai *et al. *[53,54] synthesized organic-inorganic block copolymers (PMMA*-b-*PMA and PS*-b-*PMA) containing POSS. The resulting hybrid was a well-defined, long-range structure of POSS sheets in polymer films for both annealed and unannealed films. By varying the PMAPOSS length, they were able to fine-tune the microstructures into a diverse range of morphologies, such as spheres, cylinders and lamellae, for both sets of block copolymers. The exceptional self-assembly between blocks and various morphological structures in both bulk and thin films make these POSS-based block copolymers of potential interest in block copolymer lithography.

### 2.3. POSS-Polyurethane-Based Nanocomposites

Well-characterized model systems of hybrid polyurethane-POSS (PU-POSS) nanocomposites synthesized via homogenous solution polymerization in aqueous dispersions have been reported in the literature. For example, PU-POSS nanocomposites were synthesized by incorporating amino- and hydroxyl- functionalized POSS in PU ionomeric backbones using the acetone process [29,55]. The crystallinity of the PU was found to decrease due to the incorporation of POSS. The dispersion of diamino-POSS to the hard segments of PU enhanced miscibility between the hard and soft segments, producing a more homogenous structure [55].

### 2.4. POSS-Thermosetting Polymer Based Nanocomposites

The phase behavior of POSS-capped poly(ethylene oxide) (PEO) incorporated into diglycidyl ether of bisphenol A (DGEBA) epoxy was investigated, where 4,4-methylenebis(2-chloroaniline) (MOCA) was used as a curing agent [19]. PEO-capped POSS form about 60 nm spherical aggregates in the epoxy matrix. Formation of vesicles by PEO-capped POSS was attributed to their amphiphilicity, where PEO is miscible with epoxy resins, but POSS particles are not miscible. The surface of POSS-containing epoxy resins was found to be more hydrophobic than the pure epoxy resin, as suggested by static contact angle measurements. The improvement in hydrophobicity of POSS-containing epoxy resin surface was attributed to the enrichment of the polymer surface with POSS particles. Thermal analysis of the epoxy-POSS nanohybrid revealed that none of the thermosets exhibited the melting transition of the PEO subchains, implying that the PEO chains were not crystallizable in the thermosets. The single glass transition observed indicated complete miscibility between epoxy and PEO both before and after curing [19].

**Figure 3:**
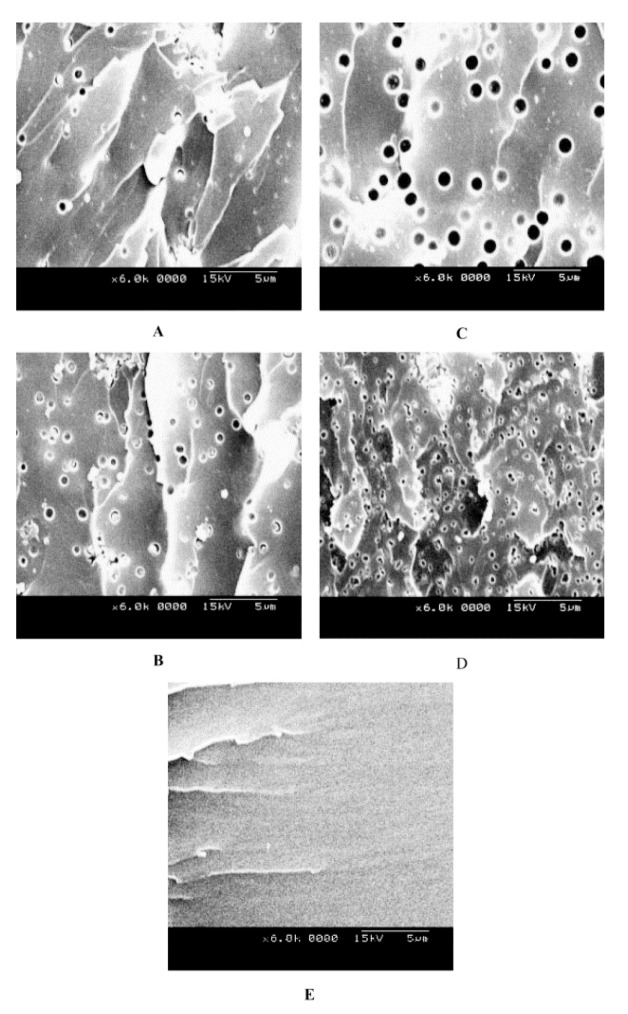
SEM images of epoxy nanocomposites containing (**A**) 5 wt.% of octanitrophenyl-POSS (ONP-POSS), (**B**) 10 wt.% of ONP-POSS, (**C**) 15 wt.% of ONP-POSS, (**D**) 20 wt.% of ONP-POSS, and (**E**) 10 wt.% of octaaminophenyl-POSS (OAP-POSS). Reproduced with permission from Ni *et al*. [56]. Copyright 2004, Elsevier.

The morphologies of POSS-containing epoxy resin-based organic-inorganic hybrids were found to be dependent on the surface functional groups of the POSS particles [56,57]. For example, incorporation of octanitrophenyl-POSS (ONP-POSS) in DGEBA epoxy resins lead to a phase separation, since ONP is not miscible with DGEBA. On the other hand, incorporation of the octaaminophenyl-POSS (OAP-POSS) nanocomposites formed a homogenous nanocomposite, as OAP is compatible with DGEBA. Scanning electron microscopy (SEM) images confirmed the difference in microstructures between both epoxy-POSS systems (see Figure 3). The epoxy/ONP-POSS hybrids exhibited heterogeneous morphology. Figure 3A–D show uniformly dispersed discrete spherical POSS-rich particles (<0.5 μm in diameter) in the continuous epoxy matrix, confirming the translucency that was observed visually. The two-phase morphologies, with a uniform dispersed domain, possess some of the familiar characteristics of phase separation induced by polymerization. Figure 3E shows the OAP-POSS-containing hybrid with no distinct morphological features, indicating a homogenous morphology. Although reference has been made to macrophase separation as being the major difference between chemically linked and physically blended polymer-POSS hybrids [14], we have presented cases of both homogenous and heterogeneous morphologies in the chemically cross-linked polymer-POSS systems. The effect of reinforcing these epoxy polymers with POSS on the mechanical properties is discussed in section 4.

### 2.5. POSS-Poly(Ethylene-Oxide)-Based Nanocomposites

The miscibility and intermolecular interactions of organic-inorganic interpenetrating polymer networks (IPNs) of octa(propylglycidyl ether)-POSS (Ope-POSS) and poly(ethylene oxide) (PEO) have been investigated by Mu *et al*. [58] A homogenous morphology was observed, suggestive of complete miscibility between the cross-linked POSS networks and the PEO in the nanohybrid. A study of amphiphilic telechelics containing hydrophilic PEO blocks and hydrophobic POSS endcaps revealed that the morphologies of the PEO crystals were affected by the level of POSS incorporation and, hence, the length of PEO block [59]. Pristine PEO consisted of spherulites. On the other hand, POSS-containing PEO formed two distinct crystalline phases: one corresponded to PEO and the other to POSS (as supported by wide angle X-ray scattering (WAXD)). PEO also was found to become less ordered due to POSS incorporation.

The influence of the surface of the functional groups of POSS on the self assembly and crystallinity of polyethylene-*block*-poly(ethylene oxide)-*block*-POSS (PE-*b*-PEO-*b*-POSS) triblock copolymers has been studied [60]. Two different types of surface functional POSS nanoparticles, isocyanatopropyldimethysilylisobutyl-POSS (ib-POSS) and isocyanatopropyldimethylsilylcyclopentyl-POSS (Cp-POSS), were used in this study. Attaching both ends of the PEO block to two other blocks caused PEO to become amorphous, while PE and POSS remained crystalline. POSS crystallization was induced by PE crystallization in the bulk PE-*b*-PEO-*b*-ib-POSS, resulting in extended chain PE crystals. In contrast, POSS molecules crystallized before the PE molecules crystallized in the PE*-b-*PEO*-b-*Cp-POSS triblock oligomer, resulting in a well-defined lamellar structure. The pre-existing POSS crystals confined the crystallization of the polyethylene molecules, causing almost once-folded PE crystals to be obtained. The confinement effect was found to play a key role on chain folding of crystalline block oligomers and, consequently, dictates their crystalline morphology. While POSS was attached to the end of the PEO block in this study [60], Zhang *et al. * [61] succeeded in incorporating POSS particles at the junction of two blocks of amphiphilic poly(styrene)-*b*-poly(ethylene oxide) block copolymer. Attachment of a POSS polymer block to poly(ethylene oxide) (PEO) made the composite polymer amphiphilic [62]. For example, POSS-PEO formed core-corona micelles in aqueous solution.

## 3. Incorporation of POSS into Polymer Matrices by Physical Blending

We have discussed that POSS can be incorporated into a polymer matrix via chemical linking with polymers and, thus, can affect structural and functional properties. However, incorporation of POSS in polymeric matrices through chemical linking involves synthetic procedures and restricts the commercialization of the resulting composite. Instead of using an *in situ* polymerization process, incorporation of POSS by using physical blending (such as melt mixing) has several advantages, such as ease of processing, versatility, being fast and cost-effective. The successful dispersion of POSS into polymeric matrices depends on the surface interactions of POSS (e.g. van der Waals, strong hydrogen bonding, and polar [14]) with polymers [33,34]. Note that POSS is available commercially with various surface functional groups (due to the advent of well developed POSS chemistry), which are advantageous in controlling POSS dispersion in polymeric matrices through cooperative surface interactions. However, interparticle interactions often result in the aggregation of POSS particles. Some representative POSS-containing polymer nanocomposites obtained by physical blending methods are presented here; we discuss the influence of POSS on the morphology of the polymer.

### 3.1. POSS-Polypropylene Nanocomposites

Incorporation of surface-functionalized POSS in polymeric matrices can influence crystallization characteristics. For example, dispersion of methyl-functionalized POSS (me-POSS) in isotactic polypropylene (iPP) increases the crystallization rate, as evidenced by differential scanning calorimetry (DSC) and small angle X-ray scattering (SAXS) analysis [63]. me-POSS particles act as a nucleating agent for iPP, and as a result, the rate of crystallization increases. In another melt-blended polypropylene (PP)/POSS hybrid, the effect of three different surface-functional POSS, methyl-POSS (me-POSS), vinyl-POSS (vi-POSS) and phenyl-POSS (ph-POSS), were studied [64]. Morphological analyses revealed that me-POSS particles form aggregates due to poor surface compatibility between polypropylene and me-POSS. The authors speculated that the observed aggregates were formed through condensation of residual silanols of me-POSS (cross-linking). At higher loadings, rod-like shape POSS aggregates were observed, and this was linked to the shear flow applied during the melt blending. SEM images for PP/vi-PSS revealed no POSS clusters at the experimental concentrations, evidence of submicron dispersion of vi-POSS within the polymer matrix. Energy dispersive spectroscopy (EDS) analyses on different areas of the sample revealed uniform silicon concentration. For the PP/ph-POSS composite, SEM micrographs showed an absence of micron-sized POSS aggregates at 1.5 and 5 wt.% POSS (inorganic) loading. However, at 5 wt.% POSS (inorganic) loading, the microstructure is not completely homogenous, as evident by some light-gray zones due to a higher local concentration of silicon [64].

### 3.2. POSS-Polystyrene Nanocomposites

Ph-POSS particles were incorporated into poly(styrene) (PS) through the solution blending method [65]. A homogenous transparent ph-POSS-containing PS nanocomposite film was obtained, indicating a uniform distribution up to 40 wt.% POSS loading. Ph surface functional groups of POSS have favorable interactions with PS, which helps to obtain uniform dispersion of POSS at such a high loading. Hydrogen bonding interactions of POSS with random thymine-PS copolymer helps the dispersion of POSS in PS-based polymers, where POSS particles were found to pack in regular order forming a crystalline domain [66].

### 3.3. POSS-Polyamide Nanocomposites

**Figure 4:**
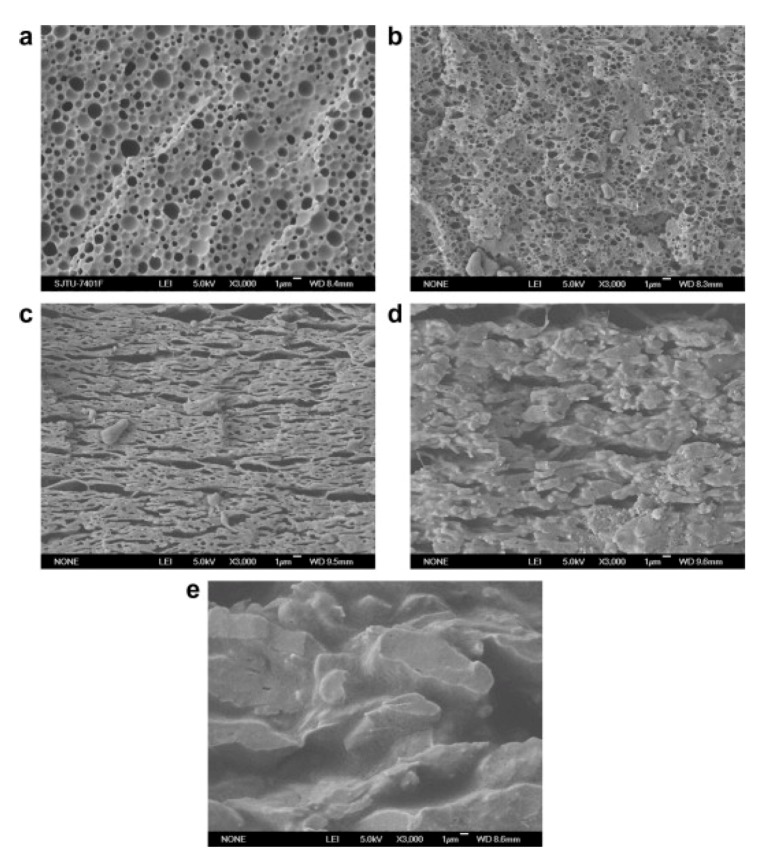
SEM images of poly(2,6-dimethyl-1,4-phenylene oxide)/polyamide (PdMPO/PA6) (50/50 phr/phr)-based polymer nanocomposites containing (**a**) 0 phr POSS, (**b**) 2 phr POSS, (**c**) 4 phr POSS, (**d**) 6 phr POSS and (**e**) 8 phr POSS. (“phr” stands for parts per 100 parts of resin.) Reproduced with permission from Li *et al*. [67]. Copyright 2009, Elsevier.

Epoxycyclohexyl-POSS was incorporated in poly(2,6-dimethyl-1,4-phenylene oxide)/polyamide (PdMPO/PA6) blends via melt mixing [67]. Morphological studies revealed a transformation from a droplet/matrix to co-continuous morphology with an increase in POSS content. The darker domains seen in the PPO/PA6/POSS SEM images (Figure 4) indicate the PdMPO phase etched by trichloromethane. PdMPO had a much higher melt viscosity than PA6 and tended to coalesce during melt-mixing. A droplet/matrix morphology was obtained for an equal amount of PdMPO and PA6 in the PdMPO/PA6 blend. The PdMPO domains as a dispersed phase have large sizes of about 0.67 μm, as seen in Figure 4a. However, at 2% POSS content, the PdMPO domain size is drastically decreased to about 0.20 μm, as seen in Figure 4b. It was concluded that the presence of POSS played an important role in decreasing the domain size of PdMPO in the PdMPO/PA6/POSS composites. This decrease in domain size was ascribed to the decrease in interfacial tension between PdMPO and PA6 in the case that the content of POSS was not very high, since highly cross-linked domains are difficult to break up. Further increase in POSS content led to a significant change in the blend morphology.

The PdMPO/PA6/POSS (50:50:4) composites had an oriented co-continuous morphology elongated along the pressure-plate direction, as shown in Figure 4c [67]. At 6% POSS content, this co-continuous morphology became rough (Figure 4d). Hence, a co-continuous morphology was obtained in a POSS-loading content, ranging from 4% to 6%. At a POSS content of 8%, the crosslinking of PA6 was said to lead to PA6 domains, with higher melt viscosity than PdMPO domains having a strong tendency to coalesce during melt mixing. Here, the co-continuous morphology was transformed back into droplet/matrix morphology, but with PdMPO domains as the continuous phase and PA6 domains as the dispersed phase, as seen in Figure 4e. Overall, the PdMPO/PA6/POSS composites with a co-continuous morphology had improved mechanical properties compared with those having the droplet/matrix morphology. We discuss the enhanced mechanical properties in detail in section 4.

### 3.4. POSS-Polyoxymethylene (POM) Nanocomposites

Polyoxymethylene (POM) is a major engineering thermoplastic commonly used to replace metal or alloy products because of its high stiffness, dimensional stability and resistance to corrosion; however, its low impact toughness and heat-resistance restricts its potentially wider range of applications [68]. The incorporation of amion functionalized POSS molecules into a POM matrix via melt-blending techniques has been demonstrated as a suitable way to improve the thermal stability of the polymer [68]. Monosilanolisobutyl polyhedral oligomeric silsesquioxane (msib-POSS) was added to POM via direct melt blending, and the morphology and thermo-mechanical behavior were studied [69]. Hydrogen bonding interactions were detected within the nanocomposites, *i.e.*, between the POM molecules and the Si-OH groups of msib-POSS. This increased the compatibility between the two components, leading to a nanosized dispersion of some msib-POSS molecules. The hydrogen bond interaction did not prevent aggregation of POSS molecules within the polymer matrix; however, it led to msib-POSS domains of micron-scale dimensions. The low temperature thermal transition and glass transition temperature of polyoxymethylene shifted to higher temperatures only for a POSS composition of 2.5 wt.%, suggesting that POSS was physically linked to POM, leading to restriction of motion under those conditions. The low content (2.5 wt.%) of msib-POSS led to antiplasticization (increase in Tg), while higher POSS content resulted in a decrease in the storage modulus of the nanocomposites relative to the neat polymer.

The effect of three different POSS, namely glycidylethyl (GE-POSS), aminopropylisobutyl (apib-POSS) and poly(ethylene glycol) (PEG-POSS) on POM was investigated [68]. The nanocomposites were produced by melt-mixing the POSS molecules with polyoxymethylene. A poor dispersion of GE-POSS and PEG-POSS molecules within the matrix was observed, as several particles between the sizes of 1 and 20 µm were observed in the micrographs. These particle sizes were due to the formation of nanospherical aggregates, indicative of phase separation in both types of nanocomposites. The sizes of the aggregates in PEG-POSS blends were larger than in GE-POSS composites, suggesting higher compatibility between POM and GE-POSS. The aggregation in the GE-POSS systems was linked to possible POSS/POSS interactions between the polymeric chain of POM and the glycidyl groups of GE-POSS. A good dispersion of apib-POSS was observed within the polymer matrix, as only a few sub-micrometer sized aggregates were obtained, evident of an almost completely homogenous microstructure. The lack of aggregates in this system suggested a high level of miscibility between apib-POSS and the POM molecules. This high compatibility was ascribed to similar polarity between amine end-groups of the POSS molecules and ether groups of the POM chain, as well as possible hydrogen bonding interactions between ether oxygen of POM and hydrogen atoms of apib-POSS. DSC analysis was used to evaluate the effect of POSS addition on the melting and crystallization behavior of the polymer. At low GE-POSS contents, the crystalline content of POM was similar to pristine POM, while at higher POSS contents, its value decreased from 58% (pristine POM) to about 53%, indicating that higher concentrations of GE-POSS slightly inhibited POM crystallization. For the POM/apib-POSS blends, the crystallinity remained fairly constant at all apib-POSS concentrations tested, while in the POM/PEG-POSS blends, the crystalline contents slightly increased, suggesting that the PEG-POSS molecules possibly acted as nucleating agents. An increase in the melting temperature of PEG-POSS molecules in the POM/PEG-POSS composites confirmed the presence of separate POSS crystalline domains. The melting temperature of polyoxymethylene in the three composites remained constant, indicating that the addition of POSS did not affect the crystalline structure of POM. These findings suggested that, while separate POSS crystalline domains representative of non-uniform dispersion were observed in the POM/PEG-POSS composites, GE-POSS and apib-POSS were well dispersed in the POM matrix, leading to the suppression of POSS crystallization.

### 3.5. POSS-Poly(Ethylene Oxide)-Containing Polymer/Block Copolymer Nanocomposites

Poly(ethylene oxide) (PEO) is a highly crystalline, water-soluble polymer with outstanding properties, such as biocompatibility and non-toxicity [62]. The effect of surface functionality of POSS on the crystallinity of PEO has been investigated by Huang *et al*. [70] The POSS molecules used in this study include octakis[dimethyl(phenethyl)siloxy]silsesquioxane (OS-POSS), octakis[dimethyl(4-acetoxyphenethyl)siloxy]silsesquioxane (OA-POSS) and octakis[dimethyl(4-hydroxyphenethyl)siloxy] silsesquioxane (OP-POSS). OP-POSS and OA-POSS were found to be completely miscible with PEO. As a result of incorporation of OP-POSS or OA-POSS into the polymer matrix, PEO was found to lose its crystallinity. OS-POSS was found to be incompatible with the PEO matrix, and therefore, OS-POSS phase separated into a PEO-rich domain and a POSS-rich domain.

**Figure 5:**
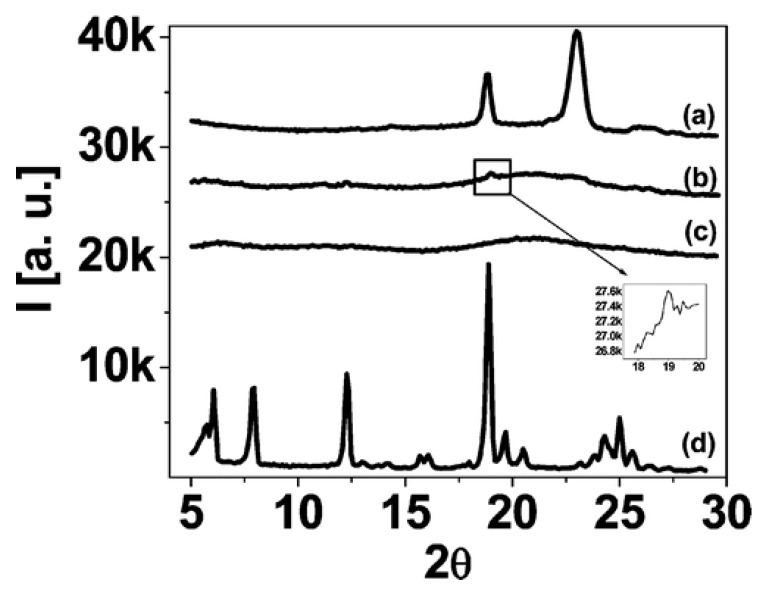
X-ray diffraction profiles of Pluronic F108 PEO-PPO-PEO block copolymer nanocomposites containing (**a**) 0 wt.% POSS, (**b**) 50% POSS–OAA, (**c**) 70 wt.% POSS–OAA and (**d**) 100 wt.% POSS–OAA. Reproduced with permission from Daga *et al.* [77] Copyright 2011, American Chemical Society.

Well-studied PEO-poly(propylene oxide)-PEO (PEO-PPO-PEO) block copolymers, known as Pluronics, are known for their versatile commercial applications. These block copolymers self-assemble into micelles [71] and various ordered nanostructures [72,73,74,75] in the presence of selective solvents. However, PEO-PPO-PEO block copolymer melts do not self-assemble because of a lack of a sufficient degree of PEO-PPO block segregation. Hydrogen bond interactions can serve as a tool for tuning self-assembly of PEO-PPO-PEO block copolymers. Daga *et al*. [76] showed that functionalized small molecules form hydrogen bonds with the PEO blocks of Pluronic block copolymers, inducing microphase segregation and, subsequently, resulting in the formation of well-ordered morphologies. Moreover, PEO-PPO-PEO block copolymers possess poor mechanical properties because of their low molecular weights. Incorporation of POSS nanoparticles (having a surface functional group that is capable of hydrogen formation) can simultaneously impart both order, as well as functional properties (such as mechanical strength). Recently, the influence of octamaleamic acid or octaaminophenyl-functionalized POSS (OAA-POSS or OAP-POSS) on the structure of Pluronic P105 (EO_37_PO_56_EO_37_) and Pluronic F108 (EO_132_PO_50_EO_132_) has been reported [77]. Both octamaleamic acid and octaaminophenyl are capable of forming hydrogen bonds with the PEO blocks. As a result of the incorporation of OAA-POSS or OAP-POSS into the polymer matrix, PEO-PPO-PEO block copolymers self-assembled into various ordered nanostructures, such as spherical, cylindrical and lamellar. Differential scanning calorimetry and X-ray diffraction (XRD) revealed good mixing of the additives within the PEO phase of the hybrid. Figure 5 compares the XRD profiles of pure Pluronic F108 and pure OAA-POSS with Pluronic F108/OAA-POSS at 50 wt.% and 70 wt.% POSS loadings [77]. Pure Pluronic F108 showed a crystalline structure resulting from the formation of PEO crystallites, while pure OAA-POSS revealed multiple peaks, an indication that crystallization occurred. After blending Pluronic F108 and OAA-POSS, the peaks observed in the neat OAA-POSS completely disappeared, suggesting molecular dispersion of OAA-POSS in the PEO phase. At 50% loading, the intensity of the peaks corresponding to PEO crystallites of neat Pluronic F108 significantly diminished in intensity, although a low intensity peak was still visible at a 2θ value of ~18. The peaks were invisible at 70 wt.% loading, suggesting complete inhibition of PEO crystallization. This implies good mixing, as the PEO and OAA-POSS inhibit each other’s crystallization associated with hydrogen bonding, rather than self-association by crystallization. The OAA ligand enabled a higher level of incorporation of the POSS particles into the PEO phase than the OAP ligand. In Pluronic F108 block copolymer strong order was maintained up to 40 wt.% and 80 wt.% of OAP-POSS and OAA-POSS loadings, respectively. OAA-POSS interacts more strongly with PEO relative to OAP-POSS, explaining the higher levels of POSS incorporation [77]. Mesoporous silica with spherical and cylindrical morphologies were obtained upon heating followed by calcination of the F108/OAA-POSS hybrid at 50 wt.% and 70 wt.% POSS loadings.

### 3.6. Other Physically Blended POSS-Homopolymer Nanocomposites

Surface functionalized POSS particles have been incorporated in many other polymeric materials to improve the functional properties of POSS-polymer composite materials. A melt mixture route was used as a means of preparing nanocomposites of high-density polyethylene (HDPE) and octamethyl-POSS (OM-POSS), and rheological studies were carried out [78]. The results revealed that, at lower filler contents (0.25–0.5 wt.%), the OM-POSS nanoparticles acted as a lubricant to reduce the viscosity of the nanocomposites, while at higher OM-POSS concentrations, there was an increase in viscosity. The POSS particles were miscible in HDPE at lower temperatures and concentrations, but formed aggregates with an increase in temperature and concentration. XRD and DSC analysis of the HDPE/OM-POSS morphology revealed that, while OM-POSS did not affect the crystallization of the high-density polyethylene, it crystallized itself into nanocrystals.

The miscibility and self-assembly of poly[(*ε*-caprolactone)-*block*-(4-vinyl pyridine)] (PCL-*b*-P4VP)-POSS blends revealed that increasing the amount of POSS in the PCL*-b-*P4VP diblock caused a change in the morphology due to increased hydrogen-bonding interactions [79]. TEM revealed spherical and cylindrical morphologies with long-range ordered structures at low POSS contents and disordered structures at relatively high POSS contents. Confined PCL phases were observed due to the pyridyl units of the P4VP block being stronger hydrogen-bond acceptors toward the OH group of OP-POSS than towards the C=O groups of the PCL block.

The morphology of PLLA/Oib-POSS nanocomposites prepared via solution casting revealed homogenous dispersion of the POSS molecules within the polymer matrix [80]. It was found that the presence of POSS significantly enhanced the crystallization rate, improved mechanical properties and accelerated the hydrolytic degradation of the polymer with respect to the control PLLA. Qui and coworkers [81] also investigated the effect of POSS hybridization of PLLA using octamethyl-POSS (ome-POSS) as nanofiller. As in the PLLA/Oib-POSS case, the ome-POSS was homogenously dispersed in the PLLA matrix. It was found that incorporating ome-POSS particles into PLLA enhanced both non-isothermal cold and melt crystallization of PLLA in the nanocomposite relative to pristine PLLA. The overall isothermal melt crystallization rate was faster in the hybrid than in neat PLLA and increased with increasing ome-POSS content.

## 4. Functional Properties of POSS-Containing Polymer Composites

### 4.1. Mechanical Properties

Incorporation of POSS in polymer matrices can enhance the mechanical properties by reinforcing polymers. The mechanical properties of POSS-containing polymer nanocomposites depend on the state of the POSS dispersion (which depends on the surface functional group of POSS), amount of POSS, crystallinity, morphology, *etc*.

**Figure 6:**
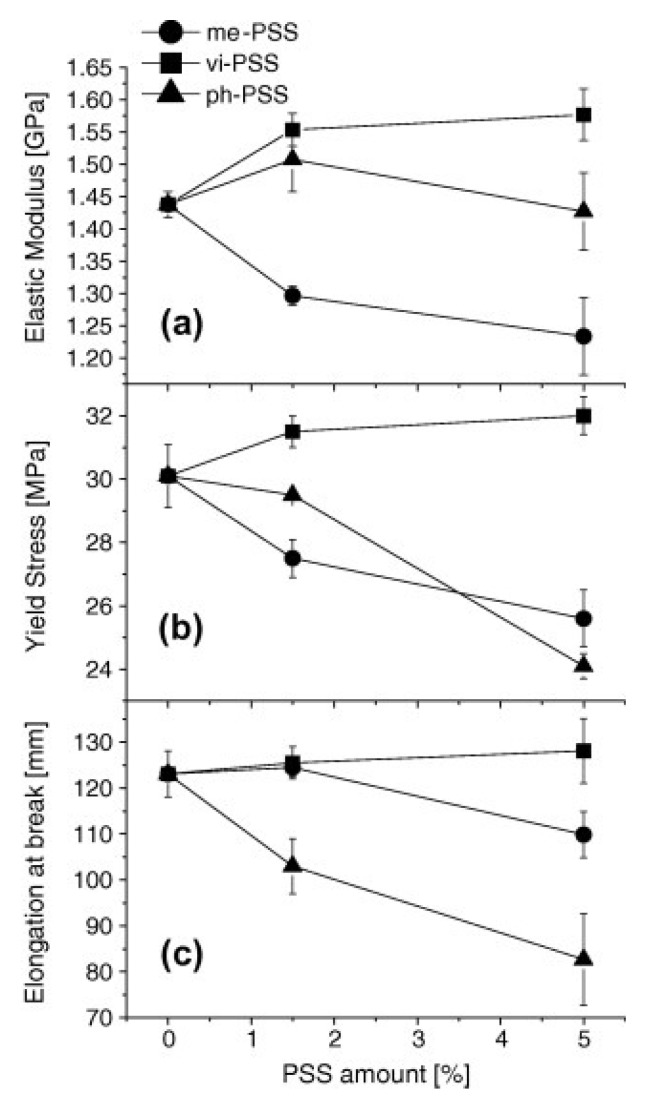
Influence of (●) methyl-POSS (me-PSS), (▲) phenyl-POSS (ph-PSS) and (■) vinyl-POSS (vi-PSS) on (top) Young’s modulus, (middle) yield stress and (bottom) elongation at break of polypropylene. Values of mechanical properties were obtained from tensile test. Reproduced with permission from Fina *et al*. [64]. Copyright 2010, Elsevier.

The influence of POSS surface functionality on mechanical properties (Young’s modulus, yield stress and elongation at break) has been studied by incorporating POSS with three different surface functional groups (me-POSS, ph-POSS and vi-POSS) into polypropylene (PP) via melt-blending [64]. Addition of vi-POSS to the polymer led to a maximum increase in the Young’s modulus, yield stress and elongation at break; hence, an overall enhancement in mechanical properties (Figure 6). This observed enhancement was ascribed to the good dispersion of vi-POSS in the polymer matrix, as well as the chemical interaction between polypropylene macromolecules and the POSS functional groups. Addition of me-POSS to polypropylene results in a moderate decrease (up to about 15% reduction at 5 wt.% POSS loading) of the Young’s modulus and yield strength values, while the elongation at break remains unaffected (Figure 6). The observed decline in mechanical properties of me-POSS-PP nanocomposites relative to neat PP was related to the presence of micron-sized POSS domains (due to aggregation of me-POSS) that behaved as weakening points during material deformation. In the case of ph-POSS containing nanocomposites. The Young’s modulus remains unaffected with increasing amount of particles, while yield strength and elongation at break were observed to decrease with increasing particle concentration (Figure 6).

Incorporation of trisilanolisobutyl-POSS was also found to enhance mechanical properties of polylactide (PLA), as evidenced by improved ductility, storage modulus and tensile strengths [82]. This property enhancement was explained by possible hydrogen bond interactions between PLA and POSS, resulting in interfacial adhesion. The storage modulus of POSS-containing PLA nanocomposites was found to decrease with increasing temperature with a significant drop in the region between 50 and 65 °C, corresponding to the glass transition region.

**Figure 7:**
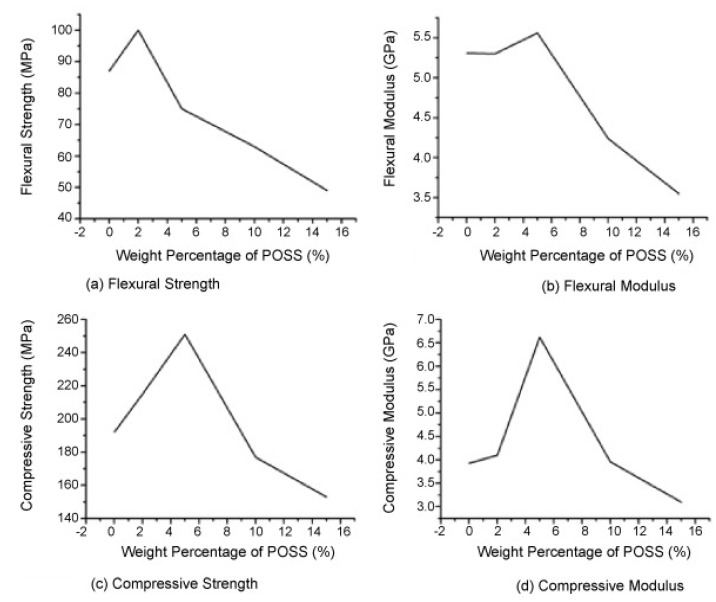
Flexural strength (top left), flexural modulus (top, right), compressive strength (bottom, left) and compressive modulus (bottom, right) of POSS-methacrylate nanocomposites are plotted *vs.* amount of POSS. Reproduced with permission from Wu *et al*. [83]. Copyright 2010, Elsevier.

The amount of POSS present can play a very important role in determining the mechanical properties of POSS-polymer composites. POSS-containing methycrylate composites could exhibit superior or inferior mechanical properties (Figure 7), depending on the amount of POSS present in the composites [83]. 2 wt.% POSS dispersion resulted in an unusual increase in mechanical properties: 15% increase in nanocomposite’s flexural strength and hardness, 12% increase in compressive strength and an unusual increase in toughness. When the POSS concentration was further increased to 5 wt.%, the compressive strength increased by 31%, while the compressive modulus increased by 68%. However, there was a decline in flexural strength from 87 MPa to 75 MPa. When the percent weight of POSS increased above 10wt%, the mechanical properties of the nanocomposites was found to deteriorate rapidly (Figure 7). The explanation behind the observed sharp decline is a very rapid formation of a cross-linked polymeric network with increasing POSS, which in turn limits the mobility of reactive species. Hence, more POSS monomers could not be polymerized, and as such, the formed aggregates consequently deteriorated the mechanical properties of the composites. This finding suggested that the reinforcing mechanism of the flexure state may be different from the reinforcing mechanism of the compressive state. Flexural strength is a very important property for dental restorations, as it reflects the ability of materials to withstand complex stress [83]. It was speculated that the uniform dispersion of the nanoparticles in the polymer matrix, as confirmed by WAXD, might be responsible for the improved properties [83]. An increase in storage modulus, tensile strength and complex viscosity of the hard segments of polyurethane films was observed with an increasing amount of diamino-POSS loadings up to 10 wt.% in POSS-containing PU polymer nanocomposites [29].

Methacrylate-based polymers have been extensively used as dental implant materials for several years [14]. Dental implants composed of methacrylate-based polymers are known [14] to exhibit toxicity, lack of strength and volume shrinkage during polymerization. Reinforcing the polymer by incorporating POSS could present a possible solution to these problems. Fong and coworkers [84] studied the effects of MA-POSS on the properties of 2,2-bis-[4-(methacryloxypropoxy)-phenyl]-propane (Bis-GMA)/tri-(ethylene glycol) dimethacrylate (TEG DMA)-based dental composites. Incorporation of small amounts of MA-POSS (*<*10 wt.%) was found to improve significantly the mechanical properties of the polymer (e.g., 20% increase in flexural strength and 35% increase in modulus). Conversely, a large amount of MA-POSS (≥10 wt.%) led to less desirable mechanical properties. By reinforcing poly(methyl methacrylate) with a small amount of MA-POSS, Gao *et al*. [85] effectively reduced the shrinkage of methacrylate-based resins and increased the double bond conversion. The POSS moieties were well dispersed in the polymer matrix, resulting in increased compressive, flexural and tensile strengths. POSS-based PMMA nanocomposites showed improved metabolic activity and lower cytolysis and mutagenesis [86].

**Figure 8:**
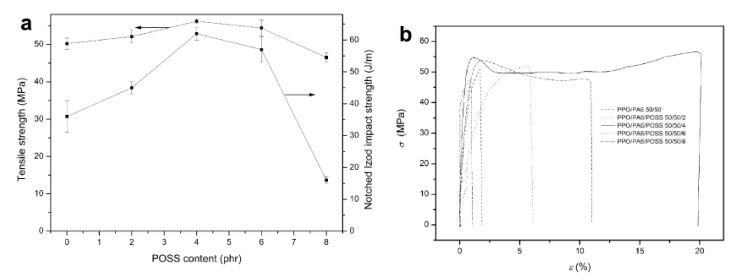
Effects of POSS on: (**a**) the tensile strength and the notched Izod impact strength; (**b**) Stress (r)–strain (e) curves of PdMPO/PA6/POSS nanocomposites. Reproduced with permission from Li *et al*. [67]. Copyright 2009, Elsevier.

The morphology of POSS-containing polymeric composites played an important role in determining the mechanical properties of the composite. For example, the incorporation of POSS into PdMPO/PA6 polymer blends was found to influence the mechanical properties of the blend by changing its morphology from droplet to co-continuous [67]. POSS-containing PdMPO/PA6 nanocomposites with co-continuous morphologies had improved mechanical properties compared with those with droplet/matrix morphologies. However, the improvement of mechanical properties of PdMPO/PA6 nanocomposites due to POSS dispersion is not continuous. For example, the tensile and impact strengths of PdMPO/PA6/POSS composites increased with increasing amount of POSS, reached to a maximum (55.2 MPa and 61 J/m) at 4 phr POSS content (Figure 8a), then decreased. From the stress-strain curves of PdMPO/PA6/POSS composites shown in Figure 8b, one could see that the PdMPO/PA6/POSS composites had higher elongation at break, with a very flexible break at POSS contents of 4 phr and 6 phr compared to 0% phr, 2 phr and 8 phr. The latter had a more brittle break without any yield point. The differences in the mechanical properties of PdMPO/PA6/POSS nanocomposites were attributed to their different morphologies.

POSS-containing polyethylene (PE) displayed superior mechanical properties than neat PE [87]. The mechanical properties of PE were found to be dependent on the degree of crystallinity [88], and therefore, a decrease in Young’s modulus of PE was expected due to the disruption of crystallinity as a result of incorporation of the bulky POSS side groups. However, it was found that the storage modulus of the PE-POSS sample (19 wt.% POSS) was fairly identical when compared with the PE control sample in a temperature range of −50 to 50 °C. This observation showed that the tensile properties were maintained irrespective of the decrease in crystallinity of polyethylene from 56% to 39%, which was due to the POSS incorporation. The presence of the POSS nanoparticles was believed to have improved the mechanical properties, thereby countering the negative effect of disrupting polyethylene crystallization.

### 4.2. Thermal Stability

Applications of polymeric materials are limited in extreme conditions because of the poor thermal stability of polymers. Incorporation of POSS in polymer matrices can improve the thermal stability of polymers because of the rigid structures of the POSS cage. The thermal stability of POSS-containing composites depends on POSS-polymer surface interactions, the amount of POSS and cross-linking. The role of POSS-polymer surface interactions on the thermal stability of the POSS-containing POM nanocomposites was investigated by dispersing apib-POSS, ge-POSS and PEG-POSS [68]. Incorporation of apib-POSS in POM was found to be effective in improving thermal stability, but not for ge-POSS and PEG-POSS. The improved thermal stability was attributed to a good dispersion of POSS in POM matrices that promoted the formation of a robust polymer/filler network.

POSS-containing epoxy nanocomposites exhibited improved thermal stabilities [56]. The surface functionality of POSS played a crucial role in improving thermal stability of epoxy-based nanocomposites. For example, OAP-POSS/epoxy nanocomposites (due to the nanoscale dispersion of POSS and the formation of a tether structure of POSS cages with the epoxy matrix) displayed a more distinct property improvement than the ONP-POSS hybrids [56]. On the contrary, incorporation of PEO-POSS was found to decrease the decomposition temperature of epoxy polymer nanocomposites [19]. Compared to control pristine epoxy, the initial decomposition of the hybrid slightly decreased with increasing PEO-POSS concentration. This decrease in initial decomposition temperature was ascribed to the incorporation of the PEO segment that possessed a lower thermal stability than the neat epoxy. Rashid *et al*. [89] reported that epoxy-POSS composites sustained higher temperatures prior to decomposition and, hence, had a higher decomposition temperature. The coefficient of thermal expansion value was also found to increase, and this was ascribed to the steric hindrance of the polymer chain due to the presence of bulky POSS side groups. Thermo-oxidative studies revealed that epoxy-POSS composites sustained higher temperatures prior to decomposition, and, hence, had a higher decomposition temperature [89].

The amount of POSS present in the POSS-polymer composites also influenced the thermal stability. Zheng *et al*. [87] studied the effect of the amount of POSS nanoparticles on the thermal stability of polyethylene (PE). POSS-PE composites exhibited an improved thermal stability (as suggested by an increase in the onset of decomposition temperature). The increase in the onset of decomposition temperature under nitrogen was attributed to possible cross-linking between scissioned PE chains and the POSS silicone core. Thermogravimetric analysis (TGA) had also suggested a significant improvement in thermal oxidative resistance in the PE-POSS composites. The improvement in thermal oxidative stability was linked to the formation of a silica layer on the surface of the polymer melt in the presence of oxygen, hence serving as a barrier and preventing further degradation of the original polymer [88]. At a 5% weight loss, the temperature of the PE-POSS copolymer is 368 °C, compared with PE control sample of 298 °C. The significant jump in temperature revealed that the incorporation of the POSS particles improved the thermal degradation behavior toward oxygen, as well as enhanced the overall thermal stability. However, this improvement decreases as the POSS concentrations exceeds 23 wt.%.

Huang *et al*. [90] studied a series of POSS/polyimide (PI) nanocomposites. The well-defined POSS particles and the strong covalent bonds between the PI and POSS led to a significant enhancement of thermal stability of the resulting nanocomposites, as a result of the significant increase of the cross-linking density in the PI-POSS nanocomposites. Wu and coworkers [91,92] synthesized POSS-containing PI nanocomposites. Solution polymerization of double-decker-shaped silsesquioxane aromatic tetracarboxylic dianhydride (DDSQDA) with several amines produced polyimides containing POSS (POSS-PI) [91]. The resulting POSS-PI hybrids demonstrated increased thermal stability relative to neat PI.

### 4.3. Glass Transition Temperature

Incorporation of POSS in polymeric matrices can alter the mobility of polymers adjacent to particles. Therefore, the incorporation of POSS particles can alter the glass transition temperature Tg of the POSS-containing composite material, which depends on several factors, such as particle-polymer surface characteristics, amount of POSS, and crystallinity of polymer.

**Figure 9:**
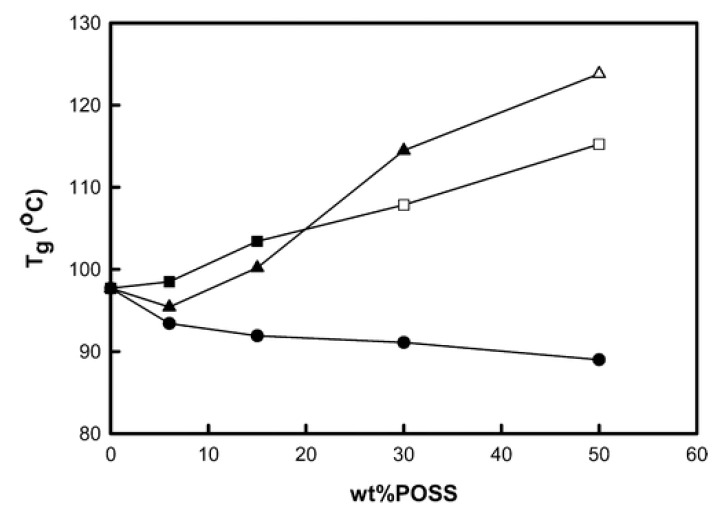
Glass transition temperature (Tg) is plotted *vs.* the amount of POSS for POSS-containing PS-based diblock copolymer nanocomposite films: (●) iBuPOSS, (■) CpPOSS and (▲) CyPOSS. Reproduced with permission from Wu *et al*. [93]. Copyright 2009, American Chemical Society.

POSS-polymer surface interactions were found to influence the Tg. The effect of three different surface functional POSS groups, isobutyl (iBu), cyclopentyl (Cp) and cyclehexyl (Cy), on the linear viscoelastic behavior of polystyrene revealed that the Tg was strongly dependent on surface functional groups of POSS particles [93]. Figure 9 shows the variation in glass transition with increasing POSS content for the three different PS-POSS systems. DSC showed that, while iBu-POSS served as a plasticizer reducing the glass transition, Cp-POSS increased the glass transition; the PS/Cy-POSS hybrids exhibited intermediate behavior. For Cy-POSS, a minimum Tg value was observed between 0 and 15 wt.% POSS loading. For a 6 wt.% Cy-POSS loading, the Tg of pure PS was higher than the polymer/POSS composite, but lower with a 15 wt.% Cy-POSS concentration. Above 6 wt.%, there was a significant increase in Tg of the Cy-POSS composites such that, above 30 wt.%, the glass transition was higher for Cy-POSS composites than for Cp-POSS composites. The variation in glass transition temperature in the polymer/POSS nanocomposites is a net result of several effects that include free volume fraction, steric barrier, as well as the polymer/POSS segment interactions [93]. As previously mentioned, a decrease in Tg was observed with increasing POSS content for iBu-POSS, indicating the POSS-segment interactions were dominated by internal plasticization of local free volume. Contrarily, the polymer-POSS segment interactions are important in determining the Tg of Cp-POSS and Cy-POSS with competition between free volume and intermolecular interactions.

To understand the effect of the amount of POSS incorporation on the Tg, Turan *et al*. [94] conducted a thermo-mechanical study on PLA-POSS and PEO-PLA/POSS. A single peak was observed in the tan δ curves of both composites, suggesting each had a single glass transition temperature. For POSS-PLA composite, at POSS loading of 1 and 3%, there was no remarkable change in the Tg of the composites; however, at 10%, a slight decrease in Tg was observed. Incorporation of POSS to the PEO-PLA had no significant effect on the Tg of the composite. The Tg of diamino-POSS-containing PU polymer nanocomposites were found to increase steadily with increasing amount of POSS [29].

Thermal studies carried out on the p(MA-POSS) system, whose morphology was described in section 3, revealed two distinct glass transitions characteristic of a microphase-separated system. Strong physical aging was observed in the microphase-separated system for annealing temperatures near the glass transition of p(MA-POSS), which was the higher Tg phase [51]. The observed aging, characterized by WAXS, suggested some level of ordering during physical aging. The Tg of the POSS-rich phase in the microphase-separated triblock copolymer was nearly 25 °C higher than in the POSS-homopolymer hybrid of similar molecular weight, indicative of Tg enhancement.

By grafting PEO onto octakis(hydridodimethylsiloxy)octasilsesquioxane (Q_8_M_8_^H^), Maitra and Wunder [95] found that crystallization was suppressed in PEO and observed an increase in Tg that was dependent on the chain length. A 35 °C increase in glass transition temperature was observed when allyl-PEO (*n* = 2) was attached to Q_8_M_8_^H^, resulting in reduced chain mobility compared to the linear material. A more dramatic effect was observed for PEO (*n* = 4), where the material turned completely amorphous from its original crystalline state, indicating four PEO repeat units were insufficient to induce crystallization at the surface. Allyl−PEO (*n* = 4) is completely crystalline, and no Tg was observed even after rapid quenching. After attachment to silsesquioxane to form Q_8_M_8_^PEO(^*^n^*^=4)^, melting was entirely suppressed, and the resulting amorphous liquid showed a Tg value quite close to the Tg obtained for *n* = 2. Since Q_8_M_8_^H^ did not exhibit a Tg before sublimation or decomposition, the authors argued that the observed Tgs resulted from the PEO segments. The attachment of PEO to the inorganic Q_8_M_8_ surface was said to restrict the mobility of the chains and suppress their crystallization. The PEO oligomers crystallized with an increasing side chain length. It is worth noting that Q_8_M_8_^PEO (*n*=2)^ and Q_8_M_8_^PEO (*n*=4)^ do not crystallize, have low Tgs and, as a result, have potential as low-temperature solvents in lithium ion battery applications [59].

### 4.4. Dielectric Properties

**Figure 10:**
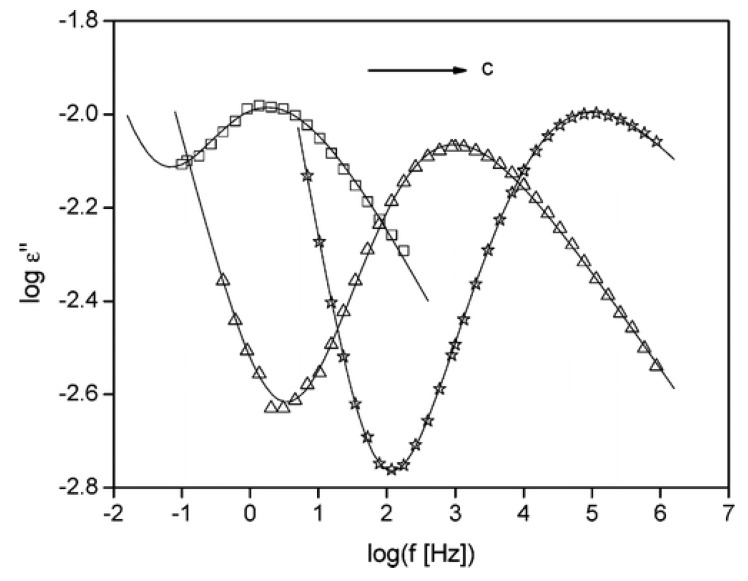
Dielectric loss is plotted *vs.* the frequency for PS-nanocomposite-zcontaining (□) 0 wt.%, (Δ) 22 wt.% and (☆) 32 wt.% POSS. Reproduced with permission from Hao *et al*. [65]. Copyright 2007, American Chemical Society.

Incorporation of nano-size filler-like POSS into polymeric matrices can improve the dielectric properties of the materials. The improvement in dielectric properties of polymer nanocomposites can be attributed to various factors, such as large particle-polymer interfacial area, particle-polymer nanoscopic structure, change in internal electric field (polarity) due to the presence of nanoparticles. The dielectric properties have been studied for POSS-containing polymeric nanocomposites using dielectric spectroscopy [65,96,97]. Figure 10 compares the dielectric loss *versus* frequency for pristine polystyrene (PS) and two PS-POSS composites at the same temperature [65]. Compared to neat polystyrene, the dielectric spectroscopy shows a strong shift to lower temperatures in the α-relaxation (related to dynamic glass transition) with increasing Ph-POSS concentrations, suggesting the POSS molecules act as a plasticizer. In addition, no other relaxation process was found in the temperature region of the glass transition of pure Ph-POSS, indicating an absence of a phase separation in the Ph-POSS/PS system up to approximately 40 wt.% of POSS. The plasticization effect of Ph-POSS was confirmed by comparing the dielectric loss *versus* frequency for pristine polystyrene and two PS-POSS nanocomposites at the same temperature. By adding 38 wt.% of Ph-POSS, the α-relaxation shifted by about sixty to higher frequencies, corresponding to a decrease in glass transition.

### 4.5. Properties Related to Biomedical Applications

POSS particles possess unique structures and superior properties that allow them to be used as fillers with polymers developed for biomedical applications, e.g., dental composites [14,86], drug delivery systems [20] and tissue engineering [98,99]. The biocompatibility of POSS because of their inert nature, as well as reduced inflammatory response, makes POSS nanoparticles very important in nanomedicine [14].

*In vivo* degradation of polyester polyurethanes over a period of six months using cross-linked POSS as the hard crystalline segments have been evaluated by Knight *et al*. [100]. No acute chronic inflammatory response was evident over a three-week period. Hence, it was concluded that the polymer/POSS composites were biocompatible. Nanocomposites for cardiovascular implants were developed by copolymerizing POSS monomers with poly(carbonate urea)urethane (POSS-PCU) [101]. POSS-PCU nanocomposite possessed unique features (such as cytocompatibility, biostability and anti-thronbogenicity), which make them suitable candidates for applications at the blood-biomaterial interface [102]. Heart valves, stent grafts and nanocomposite-coated coronary stents made from POSS-PCU nanocomposites are currently under investigation. POSS-PCU is currently being used in the design and manufacture of medical devices, such as microvascular beds for organ tissue, muscle, cartilage and breast implants, materials for coating quantum dot nanocrystals for cancer detection and many other medical applications [102].

Gupta *et al*. [103] studied biodegradable POSS-PCL-PCU and non-biodegradable POSS-PCU for use as tissue engineering scaffolds in liver, small intestine and cartilage repair, demonstrating the benefits of these composites in developing artificial organs. Scaffolds of POSS-PCL-PCU nanocomposites produced a variety of porous structures and were seeded with rat intestinal epithelial cells. The results showed that the scaffold materials supported proliferation and growth of the intestinal epithelial cells with enhanced physical and chemical properties [104]. Chemical compositions of POSS-containing nanocomposites have significant influence on the mechanical properties of tissue engineering scaffolds, as well as adhesion and proliferation of cells within the scaffold [101]. For this reason, studies have been conducted to fine-tune the chemical composition of synthetic biomaterials for improved cellular and tissue response in tissue engineering. The effects of POSS contents on the structure and biocompatibility of polyester urethane (PEU) was analyzed for the potential application of these nanocomposites in manufacturing materials, such as stents and artificial vessels, in tissue engineering [99]. The PEU/POSS nanocomposites had significant improvements in mechanical properties and degradation resistance at low POSS concentration (≤6 wt.%).

Biodegradable drug-eluting stent coatings were recently developed using thermoplastic polyurethane (TPU) with POSS hard segments (POSS-TPU) and soft segments made of poly(L-lactide)/caprolactone copolymers (P(DLLA-*co*-CL) covalently attached to PEG [20]. Highly adjustable and controllable drug release from a biodegradable stent coating was achieved. The POSS hard segments aggregated to form crystals serving as physical crosslinks on the nanometer scale, while the soft segments were designed to control the drug release rate of a mitotic inhibitor and anti-proliferative agent from the POSS-TPU stent coatings. It was shown that the hybrid polyurethane allowed a drug release rate that was controlled by variation in polymer Tg and degradation rate.

Poly(ester urethane)/POSS hybrids also supported cell growth without any toxicity recorded [98]. The concentration of POSS did not seem to affect cell adhesion or growth, however, the surface structure of PEU underwent a significant change into a three-dimensional matrix with regular pores. This allows for cells to better access nutrients, waste exchange and tissue remodeling. Degradation tests showed that the PEU/POSS hybrids were resistant to degradation over a six-month period of exposure to a buffer solution. Guo *et al*. [98] also demonstrated that the PEU/POSS hybrid had properties that supported embryonic stem cell (ESC) propagation and differentiation. These desirable properties suggest a future for these nanocomposites in tissue engineering.

## 5. Summary and Perspectives

POSS-containing polymer composite materials have attracted significant interest in recent years, as they possess nanoscopic structures and functional properties that are not typically seen in conventional hybrid materials. POSS nanoparticles are 1–3 nm in size, smaller than a polymer radius of gyration, monodisperse and rigid. Incorporation of POSS nanoparticles into both thermoplastic and thermoset polymeric matrices by chemical cross-linking or physical blending methods provides excellent reinforcement. In this review, we highlight studies on POSS-containing polymer nanocomposites that have impacted the material engineering field, with an emphasis on enhancements in mechanical, thermal stability, glass transition and biomedical properties.

Incorporation of POSS through chemical cross-linking into polymer can influence its structure, impart amphiphilicity, and improve mechanical properties by reinforcement. The POSS surface functional groups play a crucial role in controlling properties of POSS-containing hybrids. The incorporation of POSS nanoparticles into polymer through physical blending relies on favorable surface interactions between POSS and polymer. POSS having surface functional groups that have favorable surface interactions can disperse uniformly in the polymeric matrix. Uniform dispersion of POSS in polymeric matrices helps to improve physical properties of the nanocomposites. On the other hand, POSS having surface functional groups not compatible with the polymer matrix cannot be dispersed uniformly and lead to phase-separated systems with POSS-rich domain and polymer-rich domains. Such microphase separated systems cannot provide proper reinforcement and may even lead to a decrease in desired properties.

POSS-containing polymer nanocomposites are found in diverse areas, such as thermoplastics and thermosets, packaging materials, biomedicine, flow aids, surface modifiers, flame retardants, catalyst support, coatings, membrane materials, ceramic precursors, adsorbents, and dispersants [11,105,106]. Although POSS-containing polymer nanocomposites possess excellent physical properties and have the potential for wide commercial applications, large scale applications of these composite materials are limited due to high cost [107]. Over the next few years, it is possible that novel applications would be found that would potentially increase the market for POSS particles. Some promising areas for POSS include bone growth (Osteo-POSS), soft tissue healing (Fibro POSS) and flow aids for high temperature thermoplastics and catalysis of polyhedral oligomeric metallosesquioxanes [16]. While a number of significant improvements in the functional properties of the POSS-containing polymer nanocomposites have been reported, there are still a number of challenges that need to be addressed. Considerable progress has been made in controlling POSS particle distribution and location. However, there is a need to establish well-defined structure-property relationships and ways to optimize these relationships which require knowledge of the contributions of POSS nanoparticles, polymer and particle-polymer interactions to the functional properties of POSS-containing polymer nanocomposites.
